# An integrated software for virus community sequencing data analysis

**DOI:** 10.1186/s12864-020-6744-4

**Published:** 2020-05-15

**Authors:** Mingjie Wang, Jianfeng Li, Xiaonan Zhang, Yue Han, Demin Yu, Donghua Zhang, Zhenghong Yuan, Zhitao Yang, Jinyan Huang, Xinxin Zhang

**Affiliations:** 1 Research Laboratory of Clinical Virology, Ruijin Hospital, Shanghai Jiaotong University, School of Medicine, Shanghai, 200025 China; 2 State Key Laboratory of Medical Genomics, Shanghai Institute of Hematology, Ruijin Hospital, Shanghai Jiaotong University School of Medicine, Shanghai, 200025 China; 3 Key Lab of Medicine Molecular Virology of MOE/MOH, Shanghai Medical School, Fudan University, Shanghai, 200032 China; 4 Emergency Department, Ruijin Hospital, Shanghai Jiaotong University, School of Medicine, Shanghai, 200025 China; 5 Clinical Research Center, Ruijin Hospital North, Shanghai Jiaotong University, School of Medicine, Shanghai, 201821 China

**Keywords:** Virus community, High throughput sequencing, Pipeline

## Abstract

**Background:**

A virus community is the spectrum of viral strains populating an infected host, which plays a key role in pathogenesis and therapy response in viral infectious diseases. However automatic and dedicated pipeline for interpreting virus community sequencing data has not been developed yet.

**Results:**

We developed Quasispecies Analysis Package (QAP), an integrated software platform to address the problems associated with making biological interpretations from massive viral population sequencing data. QAP provides quantitative insight into virus ecology by first introducing the definition “virus OTU” and supports a wide range of viral community analyses and results visualizations. Various forms of QAP were developed in consideration of broader users, including a command line, a graphical user interface and a web server. Utilities of QAP were thoroughly evaluated with high-throughput sequencing data from hepatitis B virus, hepatitis C virus, influenza virus and human immunodeficiency virus, and the results showed highly accurate viral quasispecies characteristics related to biological phenotypes.

**Conclusions:**

QAP provides a complete solution for virus community high throughput sequencing data analysis, and it would facilitate the easy analysis of virus quasispecies in clinical applications.

## Background

Viral infections are major global public health issues and cause a high mortality rate every year worldwide. Due to the high genomic variability of RNA viruses and some DNA viruses, a massive, complex and dynamic distribution of variants, termed a viral quasispecies (QS), is generated during replication in viral infections [1]. Genetic interactions between heterogeneous mutant virus strains within a quasispecies have been proposed to affect the overall fitness of the population through a combination of cooperative and antagonistic effects, conferring high adaptability under selective pressure in changing environments, especially under the host immune response and antiviral drugs, causing immune escape and drug resistance [2, 3].

Remarkable advances in DNA sequencing technologies have enabled the comprehensive assessment of virus variability and quasispecies signatures, including the rapid evolution of next-generation sequencing (NGS) and the emergence of third-generation sequencing (TGS) [4–6]. In addition, novel sequencing strategies [6] and algorithms for virus haplotype reconstruction [7–10] are making high-throughput sequencing (HTS) preferable for quasispecies detection. Massive amounts of data have been generated, providing unprecedented opportunities to address fundamental questions in virology. However, computer-assisted technologies to determine population structure or biological functions of viruses remain a neglected area. The application of bioinformatics to this field is currently unsatisfying with respect to its medical and biological importance [11]. There are no existing tools providing a complete pipeline for quasispecies HTS data analysis and quasispecies population characteristics. Hence, an integrated and automatic software for the characterization of viral quasispecies could be of great interest for time-effective, full exploitation of quasispecies HTS data.

Viral infectious diseases include many different clinical conditions that are often not well recognized and characterized by conventional imagological and biochemical tests. Many studies have demonstrated that a viral population is highly associated with clinical manifestations and treatment responses [12–16]. Discovering biomarkers from viral quasispecies that could precisely reflect infection status has always been a pressing issue for clinicians. Therefore, discovering novel quantitative indexes to monitor virological changes is quite necessary. Reliable software with an easy-to-use interface and legible reports for viral quasispecies quantification may help with patient diagnosis, therapy, and management and eventually lead to promising advances in precision medicine in viral infectious diseases.

Here, we present QAP, an integrated quasispecies analysis package, designed with a command line utility, local graphical user interface (GUI) and cloud computation service for automatic virus quasispecies analysis. The key originality of QAP lies in not only the integrity and completeness of the analysis tools it provides but also the novel methods for quasispecies characterization and quantification. In QAP, tools for viral population quantification were developed, which provide deeper insight into quasispecies composition and a new strategy to study associations between viral populations and clinical features. QAP is freely available as a local application and as a web service to be user-friendly for bioinformatics scientists, virologists and clinicians.

## Implementation

**Fig. 1:**
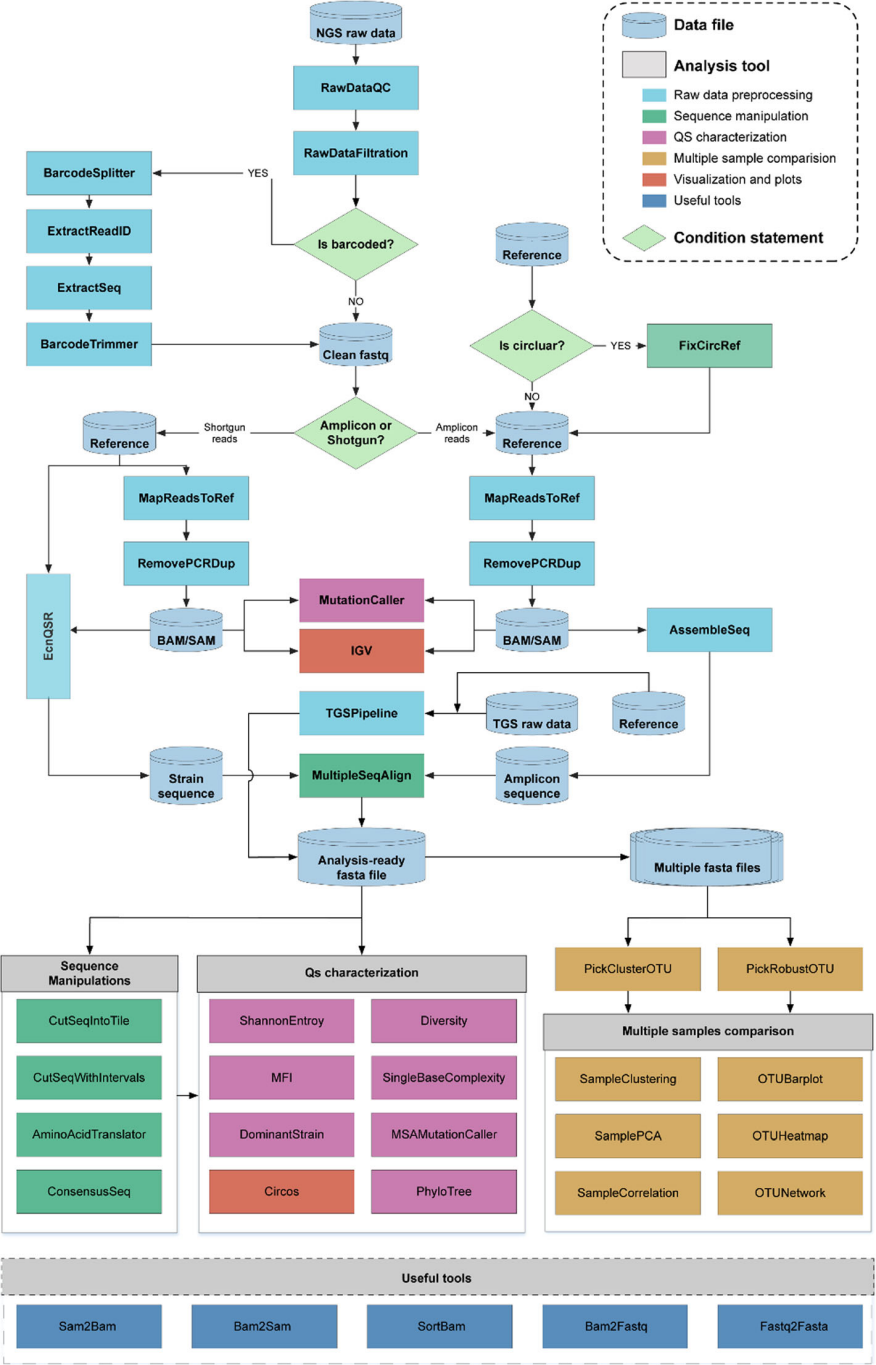
Schematic overview of tools and pipelines in QAP. Cylinders represent data files, while rhombuses represent condition statements. Rectangles represent tools in QAP, and tools in different categories are highlighted with different colours. Arrows indicate the flow between the data file, tools and condition statements

QAP is developed in Perl, R and Java and totally 41 tools were developed and categorized into 6 modules based on their functionality: (1) Data preprocessing module, (2) Sequence manipulation module, (3) Quasispecies characterization module, (4) Quantification and multiple samples comparisons module, (5) Useful tools module, and (6) Visualization module. An overview of all tools in QAP and their corresponding inputs and outputs are depicted in Additional file 2: Table S1, and the whole structure of the QAP pipeline is shown in Fig. 1. The QAP interface is facilitated through a user-friendly wrapper script, from which all tools and documentations can be invoked (Addition file 3: Fig. S1). Detailed information about each tool is available in Additional file 1.

QAP was designed to handle different kinds of sequencing data: (1) for amplicon NGS data, the tool *AssembleSeq* screens and assembles read pairs based on mapping details; (2) for shotgun NGS data, the tool *ECnQSR* includes 5 published algorithms including SAVAGE [17], ShoRAH [7], PredictHaplo [9], ViQuaS [10], and QuRe [8] to reconstruct viral haplotypes; and (3) for TGS reads, a “2-pass mapping” algorithm was developed to reads raw CCS reads generated by PacBio sequencers and processes them into aligned viral haplotypes in the tool *TGSpipeline*. The whole processing scheme of *TGSpipeline* is shown in Additional file 3: Fig. S2.

The aim of QS sequencing is to determine the precise virus spectrum, and the mapping and alignment of viral haplotype sequences should be highly accurate. Tool *FixCircRef* were designed to locate the mapping region for circular viral genomes and generate a fixed reference sequence to avoid junction mapping reads (Additional file 3: Fig. S3). Several frequently used programs for both global and local multiple sequence alignments are included in the tool *MultipleSeqAlign*, including Clustal W version 2.0 [18], MUSCLE [19] and Clustal Omega [20].

Quasispecies complexity is usually measured using normalized Shannon entropy Efficiency (Sn) according to following formula: Sn = $-\sum \limits_i\left({p}_i\mathit{\ln}\ {p}_i\right)/ lnN$ [21], where *p*_*i*_ represents the frequency of each type of strain in the quasispecies population, and *N* corresponds to the sequencing depth. In the tool *ShannonEntropy*, two methods were developed to remove bias introduced by sequencing depth: (1) use Shannon entropy instead with following formula: Sn = $-\sum \limits_i\left({p}_i\mathit{\ln}\ {p}_i\right)$, and (2) use a multiplicating random down sampling method to select a subpopulation of given size. Variation detection is crucial for quasispecies characterization. Thus, two tools *MutationCaller* and *MSAMutationCaller* were developed based on published software, including GATK [22], VarScan2 [23] and LoFreq [24]. Demonstration for software output were shown in Additional file 2: Table S2.

The MFI (Mutation frequency index) value is calculated based on the following formula: MFI = *N* / (*L* × *D*) [13, 25], where *N* represents the total number of variations detected, *L* represents the length of the amplicons and *D* represents the sequencing depth. Based on viral genomic mutations, the tool MFI can subsequently identify and visualize “hot regions” with high mutation frequencies (Additional file 3: Fig. S4A). Consensus sequences of quasispecies can be calculated by using the tool *ConsensusSeq*, which concatenates the bases with the highest frequencies at each position and provides a graphical representation of significant patterns by using WebLogo [26]. (Additional file 3: Fig. S4B). The tool *DominantStrain* calculates the proportions of different viral haplotypes and regards the highest one as the dominant strain (Additional file 3: Fig. S4C).

In order to define a unified quantitative unit, the concept of “operational taxonomic unit (OTU)” was borrowed from bacteria metagenomics analysis and re-defined here as viral strains with high homology. The tools PickRobustOTU and PickClusterOTU could define and pick viral OTUs based on sequence count (*C*) and quantity OTUs using the formula ${\mathit{\log}}_2\left(\frac{C}{N}M+1\right)$, where *C* represents the sequence count of a specific OTU, *N* represents the total number of sequences, and *M* represents a multiplier coefficient that corrects the minimum $\frac{C}{N}$ into a positive float more than 1. OTU abundance matrix were then normalized by using R package preprocessCore. The workflows of *PickRobustOTU* and *PickClusterOTU* are shown in Additional file 3: Fig. S5.

### Cloud computation platform

We developed a web-based computation platform for QAP, named wQAP. wQAP was built on top of Galaxy [27] which was constructed by using Django framework.

**Fig. 2:**
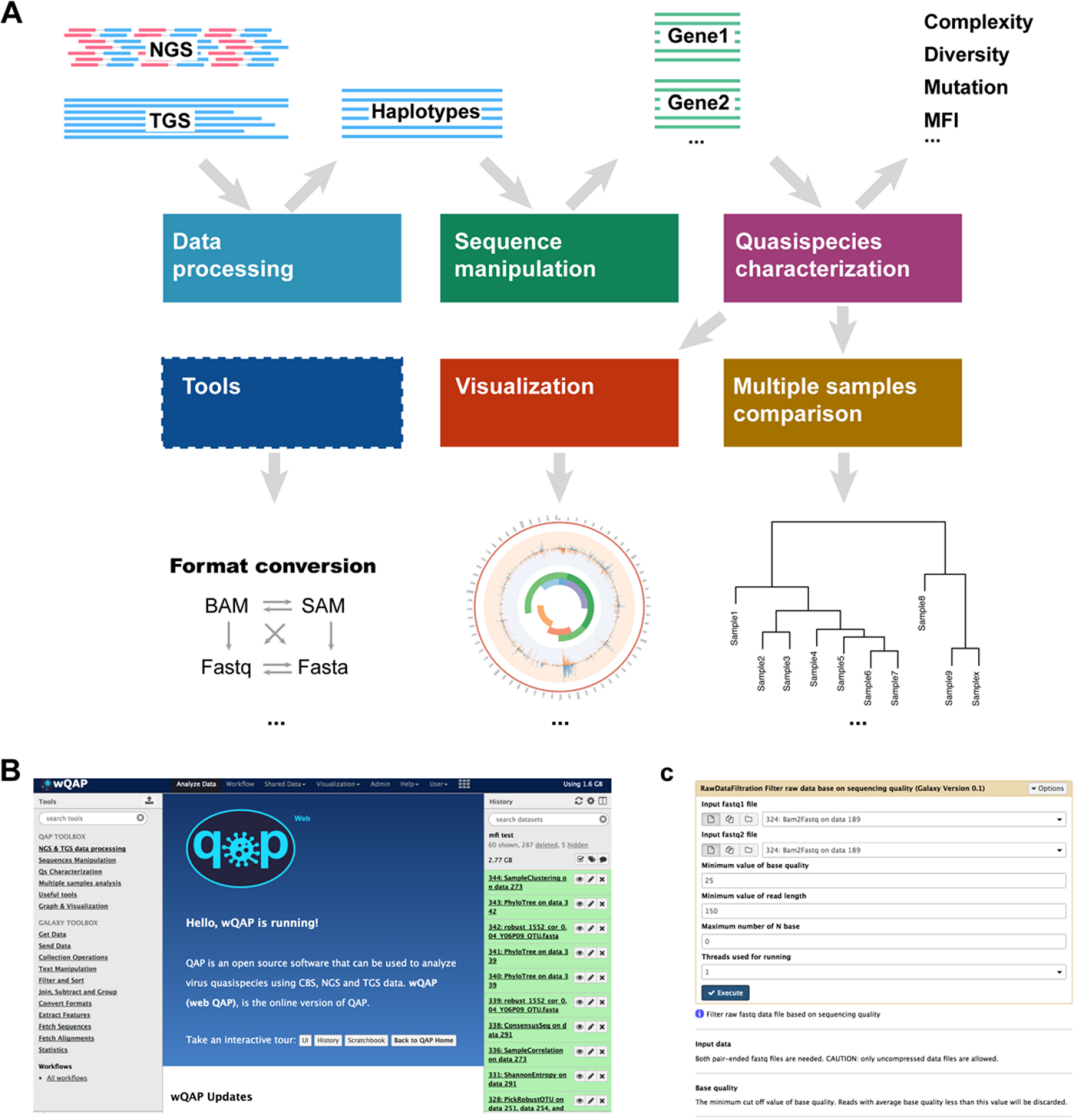
Workflow and screenshots of wQAP. **a** Workflow of wQAP. Coloured rectangles correspond to six tool categories. Lines represent data files, including input sequencing reads, viral haplotypes and viral genes. Arrows indicate the flow between inputs, processes and outputs. **b** Screenshot showing the main page of wQAP. **c**, Screenshot showing usage of the tool *RawDataFiltration*

When using wQAP, raw data will be added to the user history and processed by analysis modules step-by-step (Fig. 2a). As shown in Fig. 2b, c, all tools can be easily accessed from the main page, including both QAP tools and tools embedded in Galaxy. To support the Workflow Management System of Galaxy, tools in wQAP are also designed with optimized input and output format, which could be easily connected and constitute customized pipelines.

### Local graphic user interface

**Fig. 3:**
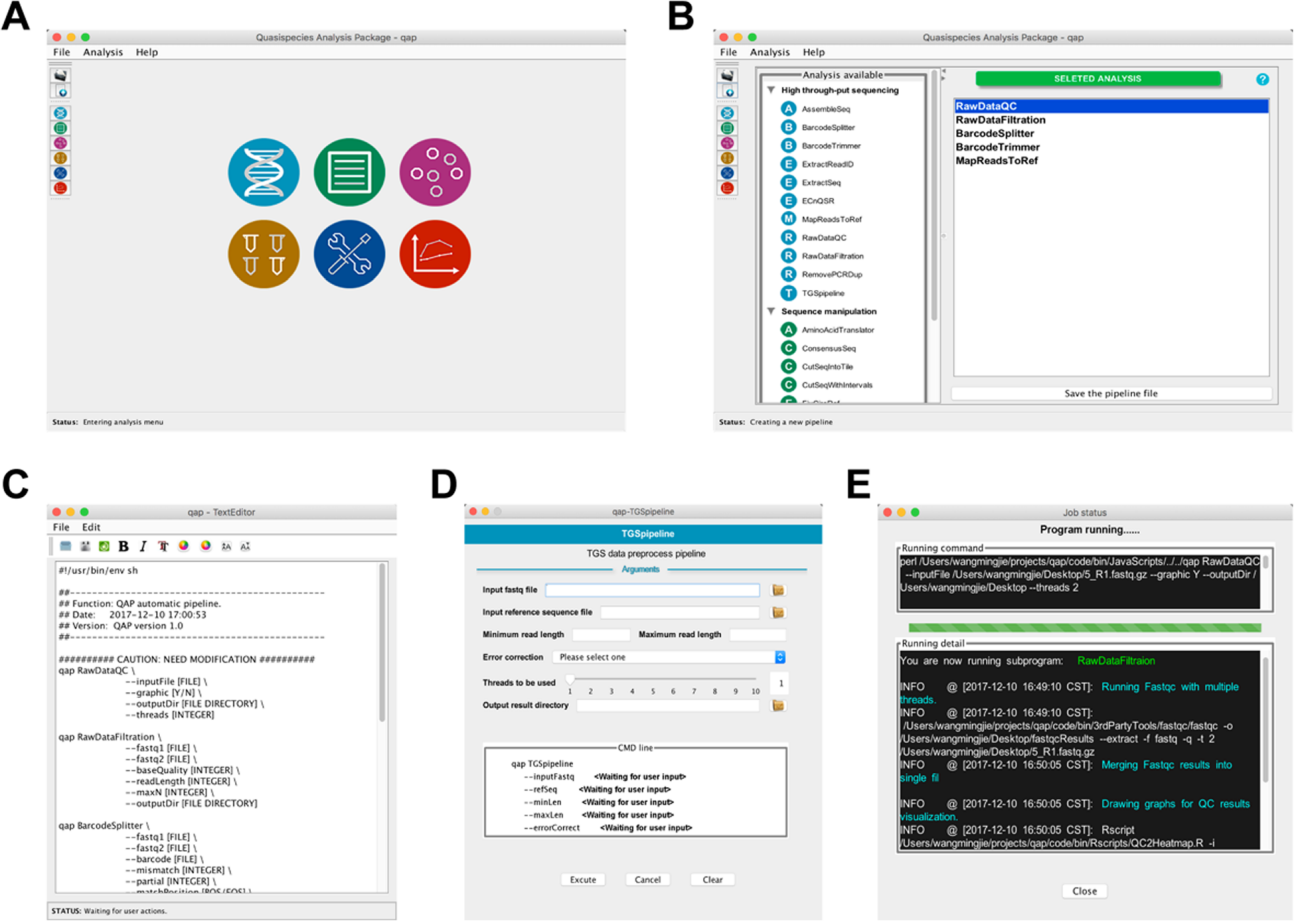
Schematic overview of pipeline construction and screenshots of the QAP GUI. **a** Screenshot of the QAP GUI main interface. **b** Screenshot showing the pipeline construction interface. **c** Screenshot showing the pipeline Shell script generated. **d** Screenshot of the argument input interface. **e** Screenshot of the program running interface

The QAP GUI is implemented in Java as a desktop application which could be activated by using “qap –g” in command line. Screenshots of the QAP main interface and pipeline construction interface are shown in Fig. 3a, b. The GUI application generates a JSON file to save the customized pipeline structure and an executable shell script for direct usage (Fig. 3c). As shown in Fig. 3d, arguments can be provided through text fields or drop-down lists. After checking the validity of arguments, the program will start running and representing output information (Fig. 3e).

## Results

A broad range of tools were developed in QAP for users to analyse data from different angles and build customized pipelines. Below, we evaluate the utilities of QAP using different kinds of virus community sequencing data.

### Comprehensive evaluation using HBV quasispecies sequencing data

To assess the advantages of integrated analysis tools in QAP, comprehensive data sets of HBV QS were used, including clone-based sequencing (CBS), NGS and TGS data. Datasets details were described in Additional file 1, and amplification details for HBV NGS data were also illustrated in Additional file 2: Table S3, and Additional file 3: Fig. S6.

As CBS is considered the “gold standard” in quasispecies detection [28, 29], paired TGS and CBS data derived from 10 HBV-infected patients were analysed to measure the accuracy of QAP in TGS data processing. Bland-Altman approach was carried out to compare the quasispecies heterogeneities of 4 viral ORFs (C, P, S and X) derived from TGS and CBS, and the result indicated a high level of agreement (Additional file 3: Fig. S7).

**Fig. 4:**
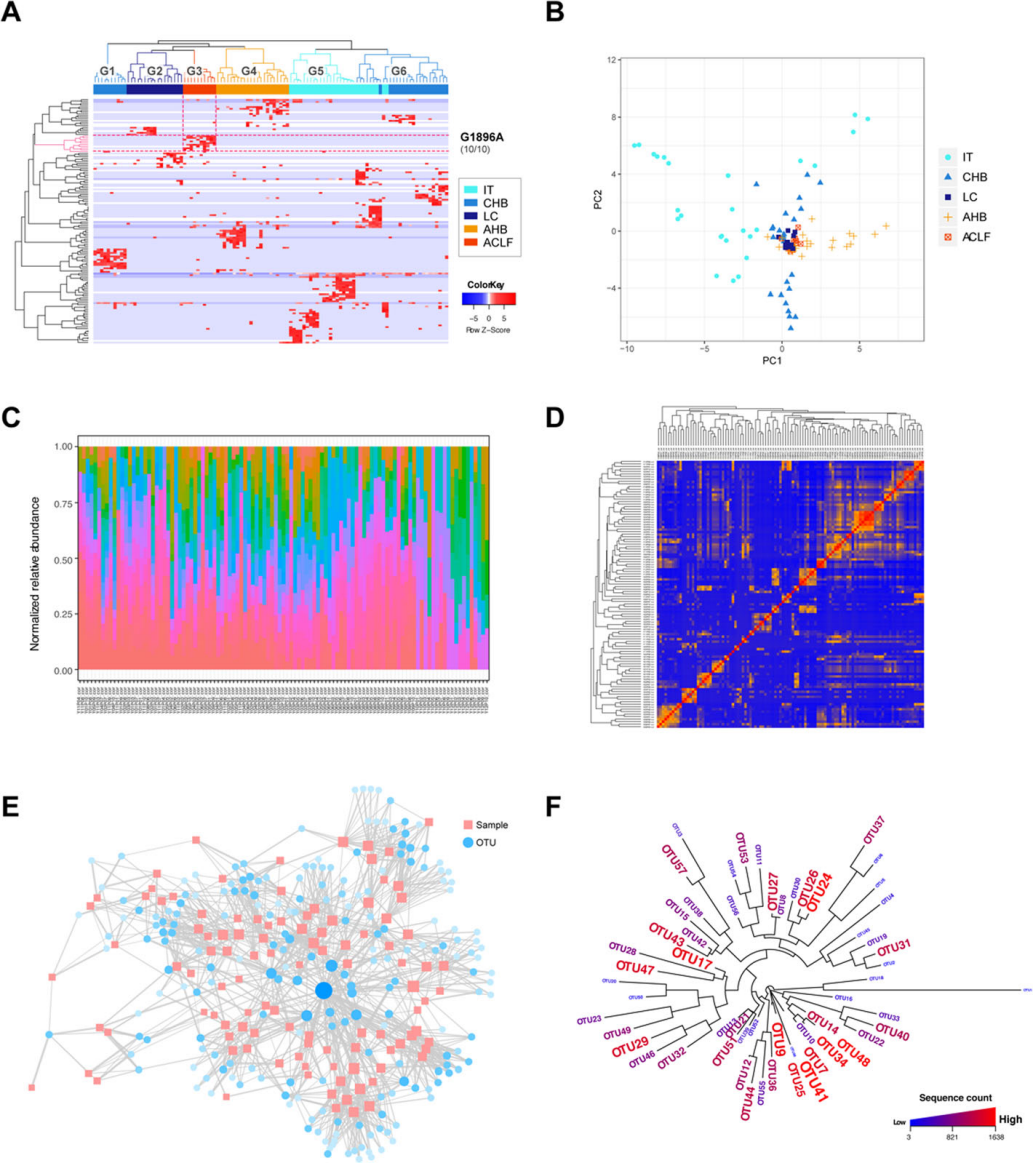
Example outputs from QAP analysis of NGS of HBV QS data. **a** Hierarchical clustering of samples and OTUs. Representative OTUs corresponding to ACLF patients are highlighted with red lines. **b** Scatter plot showing the PCA results. **c** Bar plots showing OTU abundances. **d** Heat map showing sample correlations. **e** Network showing correlations between samples and OTUs. Node size and colour correspond to OTU abundance and sample weight. **f** Phylogenetic tree showing OTU homology; font size and colour corresponds to OTU abundance

To further explore the functionality and clinical significance of QAP tools, a retrospective cohort study analysing an HBV whole-genome quasispecies was carried out. Clinical features of all patients are summarized in Additional file 2: Table S4. Hierarchical clustering analysis was carried out to explore the correlations between viral populations and clinical phenotypes. Notably, the dendrogram of patients generated by OTUHeatmap showed significant clusters (subgroups G1-G6) correlated to infection phases (Fig. 4a, *P* = 2.20 × 10^− 16^), and PCA carried out by SamplePCA showed similar results (Fig. 4b, *P* = 1.35 × 10^− 13^). Furthermore, sample clustering and the top 3 principle components all showed significant correlations with patients’ clinical traits (Additional file 2: Table S5). Viral spectrum structures of different samples were also explored by using OTUBarplot (Fig. 4c), and distinct components were discovered. Correlations among different samples were also analysed by using SampleCorrelation (Fig. 4d). A network among different samples and OTUs was constructed, and significant OTUs were highlighted (Fig. 4e). Phylogenetic analysis was also carried out based on OTU sequences (Fig. 4f).

### Evaluations using various viral community sequencing data

**Fig. 5:**
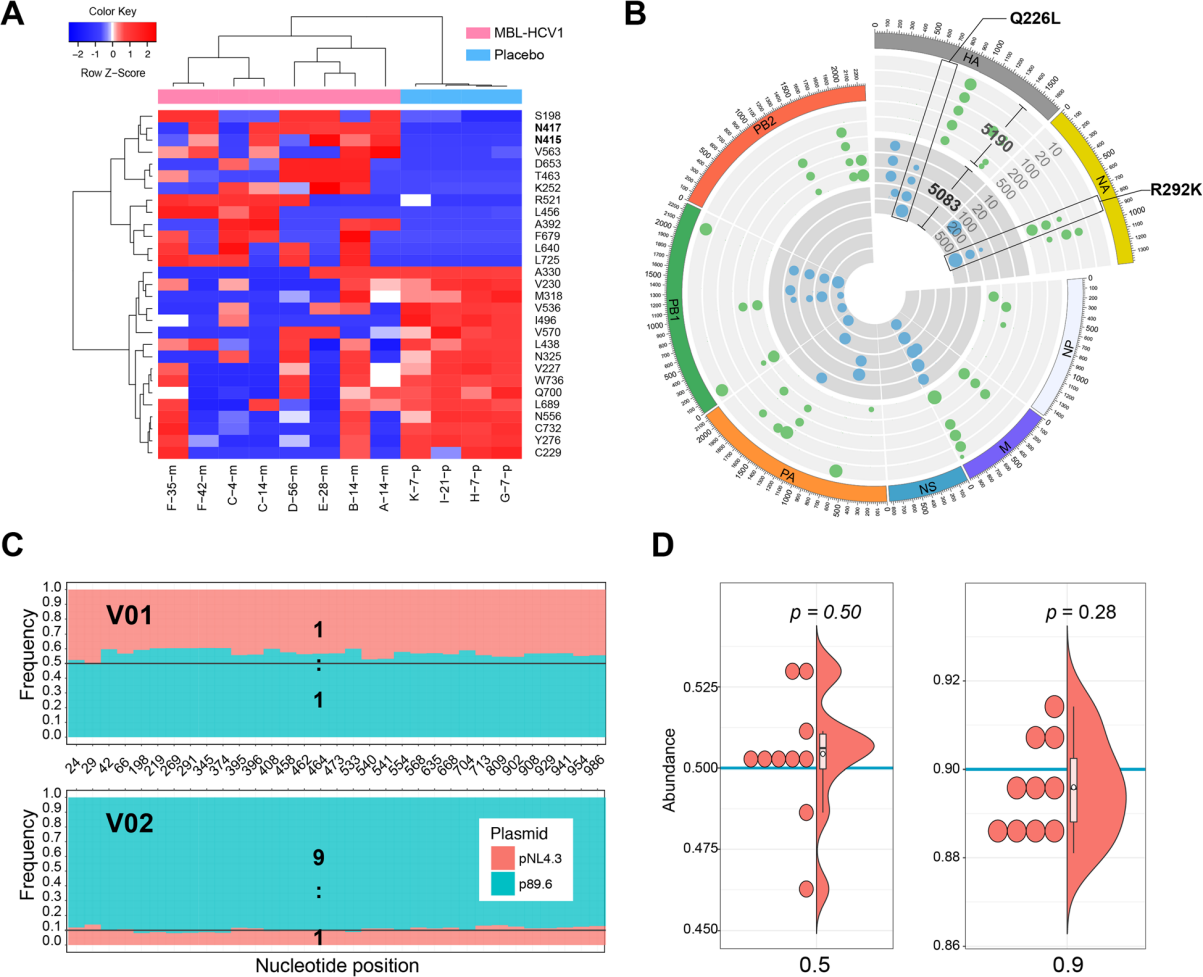
Outputs from QAP analysis of HCV,H7N9 and HIV QS data. **a** Hierarchical clustering of mutation sites in the HCV E2 region. **b** Circos plot of mutations in two H7N9 strains. The outside track corresponds to 8 segments of the viral genome. Tracks in the light grey (dark grey) background colour correspond to strain 5109 (5083) treated with 10 μM, 20 μM, 100 μM, 200 μM and 500 μM oseltamivir. Green (blue) dots represent variations in strain 5109 (5083), and dot size corresponds to mutation frequencies. **c** Frequencies of mutation sites between pNL4.3 and p89.6 in the mixtures V01 and V02. Bar height corresponds to base abundance. The true ratios are marked with black lines. **d** Half violin plot of abundances of HBV genotype C viral strain in simulation data sets. The true ratios are marked with blue lines

QAP utilities were further tested on different viruses, including HCV, H7N9 and HIV and simulated data of HBV. Shot-gun sequencing data of HCV was derived from the study of Babcock G.J. et al. [30], in which HCV E2 region of 6 antibody (MBL-HCV1)-treated subjects and 5 placebo-treated subjects were sequenced. Mutations in all subjects were identified, and showed consistent results with previous study (Fig. 5a, Additional file 2: Table S6) [30].

Two human H7N9 strains (5190, 5083), which were isolated from two infected patients [31, 32], were cultured in ascending concentrations of oseltamivir carboxylate with the help of exogenous neuraminidase to induce oseltamivir resistance mutations. Details for H7N9 amplification were described in additional file 1 and additional file 2: Table S7. The results showed that frequencies of the drug resistance mutation Q226L and R292K elevated gradually with increasing concentrations of oseltamivir carboxylate. (Fig. 5b). Amplicon sequencing data from mixtures of two HIV plasmids were retrieved from the NCBI SRA database. Mixture V01 consisted of 50% plasmid pNL4.3 and 50% p89.6, while mixture V02 consisted of 10% pNL4.3 and 90% p89.6. The abundance of viral strains were highly consistent with mixing proportions (Fig. 5c). All of these results confirmed the effectiveness and practicability of QAP in viral community sequencing data analysis. We further evaluated the performance of QAP with two groups of simulation data sets which were generated with pre-defined abundances of viral strains, and the result also showed high consistency between observed values and true values (Fig. 5d).

### Comparison of QAP with existing tools

We compared QAP with several existing software platforms, including SAVAGE [17], ShoRAH [7], PredictHaplo [9], QuRe [8] and ViQuaS [10], to investigate their calculation performances. All software was tested using HBV TGS and NGS data, and QAP and ViQuaS demonstrated the best time efficiency when testing NGS data, while QuRe was the most time-consuming, which was consistent with published results [10, 33]. A summary of the specialties of QAP and other existing software were shown in Additional file 2: Table S8.

### Clinical applications of QAP quantitative methods

**Fig. 6:**
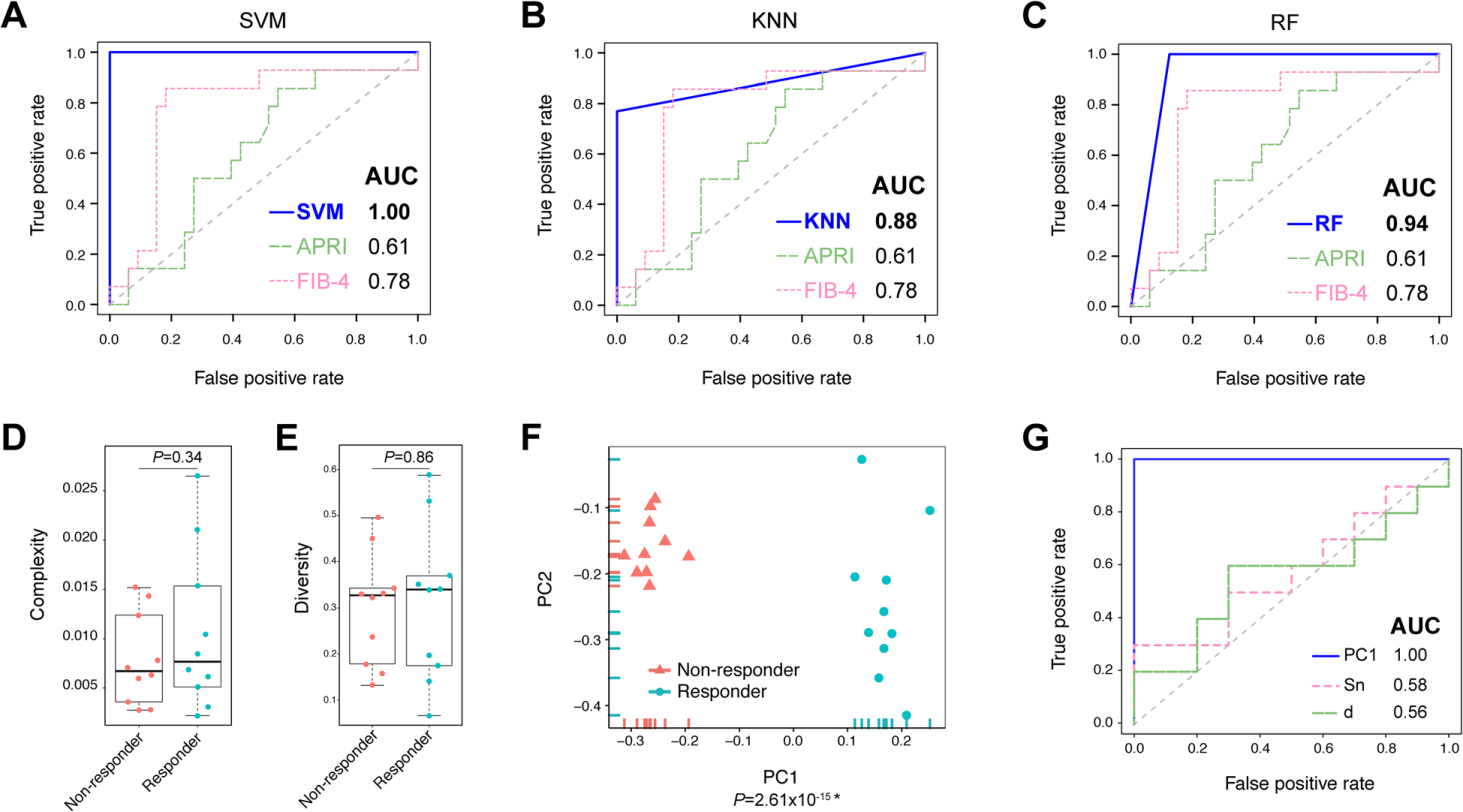
Performance of novel diagnostic and predictive models based on QS quantification. **a**-**c** Receiver operating characteristic (ROC) curves of LC diagnostic models, including SVM, KNN and RF models. **d**-**e** Sn and d between QS of responders and non-responders. **f** PCA of QS of responders and non-responders. **g** ROC curves of PC1 compared with Sn and d

The clinical applications of OTU quantification were further evaluated in chronically HBV-infected patients, including LC patients and non-liver cirrhosis (NLC) patients. To build diagnostic models for LC patients based on viral population quantification, both LC and NLC patients were randomly and equally divided into training groups and validation groups. Three diagnostic models were built by using machine learning methods, including support vector machine (SVM), K-nearest neighbour (KNN), and random forest (RF), based on viral strain abundances of patients in training groups. The performances of all models were then evaluated and compared to the commonly used clinical parameters APRI and FIB-4 in validation groups. The results showed that the SVM model had the highest accuracy for LC patient diagnosis with an AUROC (area under receiver operating characteristic curve) value equal to 1.00 (Additional file 2: Table S9, Fig. 6a, b, c).

Quasispecies between 10 antiviral therapy responders and 10 non-responders were compared at treatment baseline. QS heterogeneities, including Sn and mean genetic distance (d), both showed no significant difference between two kinds of patients (Fig. 6d, e). However, by using QAP quantification methods, PCA of QS showed significantly separated clusters, and principle component 1 (PC1) was significantly related to treatment outcomes (*P* = 2.61 × 10^− 15^) (Fig. 6f), which showed superior performance in distinguishing responders and non-responders relative to general quasispecies heterogeneities (Fig. 6g).

## Discussion

Viral haplotype determination is the key step in virus community data analysis. There are several possible approaches for determining virus haplotypes: (1) pair-ended amplicon sequencing with relative long read length [34]; (2) quasispecies reconstruction based on shotgun sequencing [7–10]; (3) single molecule sequencing platforms, such as PacBio [28, 35] and Nanopore; and (4) barcode-tagged refined sequencing methods [6]. QAP is the first all-in-one software for virus community data analysis that meets the requirements for various sequencing platforms and strategies.

The key innovation of QAP is the development of viral OTU quantitative methods, which allow exploration of a new field for virology research. In chronically HBV-infected patients, the early diagnosis of liver cirrhosis is crucial for making treatment decisions. Here, we preliminarily studied the clinical usage of QAP and built diagnostic models based on viral OTU quantification. Thus, QAP might promote the application of quasispecies in clinical practice and shed light on precise diagnosis in countless virus-infected patients.

Recently, Docker has received increasing attention throughout the bioinformatics community. A Docker image with QAP and all dependencies was developed, allowing the straightforward use of QAP on any operating system. Thus, totally four distribution forms of QAP are provided to meet the needs of different users: a command line program, a Galaxy-based web server, a GUI program and a Docker image.

## Conclusions

we present QAP, an integrated application and web service for virus community sequencing data analysis. QAP allows comprehensive and rapid characterization of quasispecies from different platforms and sequencing strategies, which have been demonstrated using HBV, HCV, HIV and H7N9-related viral community studies. In addition, QAP was first implemented for quasispecies quantification among multiple samples, facilitating the discovery of important correlations between the virus spectrum and clinical phenotypes in HBV-infected patients, showing great potential in patient diagnosis and prognosis prediction. The QAP web application and local GUI facilitate the easy analysis of virus quasispecies by clinicians and other laboratories. We expect that QAP will be a starting point for researchers to dive more deeply into computational analysis in relevant fields, and finally accelerate the application of quasispecies in routine clinical tests in the future.

## Availability and requirements

- Project name: QAP
- Project home page: http://life2cloud.com:6005/qap
- Operating system(s): Platform-independent
- Programming language: Perl, R, Java
- License: All software and scripts are licensed under GPLv3.

## Supplementary information

**Additional file 1 Supplementary Methods.** Detailed methods for pipeline implementation and generation of data for software evaluations.

**Additional file 2 Table S1.** An overview of tools in QAP. **Table S2.** Demonstration of outputs of tools *MutationCaller* and *MSAMutationCaller*. **Table S3.** Forward and reverse primers for HBV genome amplification. **Table S4.** Characterizations of clinical features of HBV infected patients. **Table S5.** Statistical significance of associations between sample subgroups/principle components and clinical traits. **Table S6.** Drug resistance associated mutations of all subjects in HCV shot-gun sequencing data analysis. **Table S7.** Forward and reverse primers for H7N9 genome amplification. **Table S8.** Overview comparison of QAP and existing software. **Table S9.** Performance of LC diagnostic models and clinical parameters.

**Additional file 3 Figure. S1.** Screenshot of the QAP main program in command line. **Figure S2.** Schematic overview of the tool *TGSpipeline*. **Figure S3.** Schematic overview of the tool *FixCircRef*. **Figure S4.** Example results generated by QAP. **Figure S5.** Schematic overview of OTU picking. **Figure S6.** Example results generated by Circos and IGV tools showing the amplicons of HBV whole-genome sequencing. **Figure S7.** Bland-Altman analysis of heterogeneity of 4 ORFs in TGS data compared with CBS data.

## Data Availability

All releases and detailed documentation of QAP, web service wQAP and simulation data can be found at http://life2cloud.com:6005/qap. A release version of the QAP source code can also be found at Github https://github.com/mingjiewang/qap/. Docker images are deposited at Docker hub https://hub.docker.com/r/mingjiewang/qap/. HBV CBS raw data have been deposited at NCBI GenBank under accession IDs KY881721 to KY882003. HBV NGS, HBV TGS, H7N9 NGS raw sequencing data have been deposited at the NCBI Sequence Read Archive (SRA) under accession IDs SRP126807, SRP128001 and SRP139718. HCV and HIV NGS data were downloaded from NCBI SRA under accession IDs SRP037575 and SRP132841.

## Abbreviations

ACLF: Acute-on-chronic liver failure
AHB: Acute hepatitis B
CBS: Clone-based sequencing
CHB: Chronic hepatitis B
GUI: Local graphical user interface
HTS: High-throughput sequencing
IT: Immune tolerance
KNN: K-nearest neighbour
LC: Liver cirrohosis
MFI: Mutation frequency index
MSA: Multiple sequences alignment
NGS: Next-generation sequencing
NLC: Non-liver cirrhosis
OTU: Operational taxonomic unit
PCA: Principle components analysis
QAP: Quasispecies analysis package
QS: Quasispecies
RF: Random forest
Sn: Shannon entropy Efficiency
SVM: Support vector machine
TGS: Third-generation sequencing
