# Supplementary material for: An integrated software for virus community sequencing data analysis
Source: BMC Genomics. 2020 May 15;21:363. doi: 10.1186/s12864-020-6744-4 (PMC7227348; doi:10.1186/s12864-020-6744-4)
Supplement: Supplementary file 1 — Additional file 1 Supplementary Methods. Detailed methods for pipeline implementation and generation of data for software evaluations. [file 12864_2020_6744_MOESM1_ESM.docx]

**Supplementary Methods**

**Pipeline implementation**

There were totally 41 tools developed and categorized into 6 modules based on their functionalities.

**Data preprocessing module**

The tool *RawDataQC* uses FastQC [1] to check the quality of input fastq files. Cutadapt is used for reads filtration in the tool *RawDataFiltration* with two main parameters regarding base quality and read length. The tools *BarcodeSplitter* and *BarcodeTrimmer* are designed for barcode identification and trimming by using regular expression in Perl. The tools *ExtractReadID* and *ExtractSeq* were developed in R. In the tool *MapReadsToRef*, two commonly used programs, BWA mem [2] and Bowtie2 [3], are provided for mapping short reads to a reference genome, with the parameters “--very-sensitive-local” enabled when Bowtie2 is used. For PCR duplicates removal, Picard tools and SAMtools [4] are used by the tool *RemovePCRDup*. The tool *AssembleSeq* extracts read pairs from a BAM file and assembles them according to overlapped bases. For error correction and shotgun sequencing quasispecies reconstruction, 5 programs are used in ECnQSR: SAVAGE[5], ShoRAH [6], PredictHaplo [7], ViQuaS [8], and QuRe [9]. In the tool *TGSpipeline*, raw reads are first mapped to a reference sequence by using BLAST [10], and then fragments with high bit scores are extracted and mapped back to the reference genome to replace sequence fragments at the same location. Sequence variants are identified and classified into SNVs, insertions, high-confidence (HCon) deletions and low-confidence (LCon) deletions and then passed to ShoRAH [6] for error correction. The whole processing scheme of TGSpipeline is shown in Additional file3: Fig. S2.

**Sequence manipulation module**

The tool *FixCircRef* first extends both 5’ and 3’ ends of a raw reference sequence by 500 bp and uses Bowtie2 to map short reads to the extended sequence, then calculates reads coverage and depth by using SAMtools [4] and finally identifies the actual start and end position to generate a fixed reference sequence. The processing pipeline is shown in Additional file 3: Fig. S3. Clustal W version 2.0 [11], MUSCLE [12] and Clustal Omega [13] are used for multiple nucleotide or amino acid sequences alignment in the tool *MultipleSeqAlign* with their respective default settings and parameters, which are non-configurable. Consensus sequences of quasispecies can be calculated by using the tool *ConsensusSeq*, which concatenates the bases with the highest frequencies at each position and provides a graphical representation of significant patterns by using WebLogo [14].

**Quasispecies** **characterization module**

Quasispecies complexity is usually measured using normalized Shannon entropy Efficiency (Sn) according to following formula: Sn = $-\sum_{i} (p_{i}ln p_{i})/lnN$, where *p_i_* represents the frequency of each type of strain in the quasispecies population, and *N* represents the total number of strains, which corresponds to the sequencing depth. In the *tool* *ShannonEntropy*, two methods were developed to remove bias introduced by sequencing depth: (1) use Shannon entropy instead of normalized Shannon entropy efficiency with following formula: Sn = $-\sum_{i} (p_{i}ln p_{i})$, and (2) use a multiplicating random sampling method, which allows users to select a subpopulation of quasispecies with a given population size for specific replications and then calculate the mean value. All calculations are implemented by using R scripts. The tool *Diversity* classifies input sequences into 3 types (non-coding nucleotide sequence, coding nucleotide sequence and amino acid sequence) by using the argument “--seqType” and then calculates 3 indexes for sequence diversity, i.e., d (mean genetic distance), dS (number of synonymous substitutions per synonymous site), and dN (number of non-synonymous substitutions per non-synonymous site), by using MEGA CC 6.0 [15]. The tool *SingleBaseComplexity* calculates the Sn at each site of the quasispecies sequence by first cutting sequences into single bases and then invoking the tool *ShannonEntropy* for Sn calculation. The tool *MutationCaller* integrates three popular programs for detecting variations, including GATK v3.7 [16] (both HaplotypeCaller and UnifiedGenotyper algorithms), VarScan2 [17] and LoFreq [18]. *MutationCaller* can read BAM files and output variations in the VCF format. *MSAMutationCaller* reads multiple sequences alignment (MSA) data and identifies and filters bases that are different from reference sequences and output variation sites and frequencies. The tool *MFI* was developed to highlight highly variable regions based on variations detected by *MSAMutationCaller*. The MFI (Mutation frequency index) value is calculated based on the following formula: MFI = *N* / (*L*×*D*), where *N* represents the total number of variations detected, *L* represents the length of the amplicons and *D* represents the sequencing depth. The tool *MFI* runs in two modes: (1) with the parameter “--mode seq”, the MFI of the whole amplicon is calculated, and (2) with the parameter “--mode span”, the program cuts sequences into short spans and then calculates the MFI of each span. The tool *DominantStrain* calculates and visualize the proportion of viral strains using R scripts.

**Quantification and multiple samples comparisons module**

The concept of “operational taxonomic unit (OTU)” was first borrowed from bacteria metagenomics analysis and re-defined here as viral strains with high homology. Tools *PickRobustOTU* and *PickClusterOTU* read virus haplotype sequences in fasta format, quantify each viral strain of each quasispecies and then filter strains with high frequencies. After strain filtration, a summary table of all strains in each sample will be generated, and *PickRobustOTU* will pick strains with high ratios and rename them with a numeric sequential OTU ID. Unlike *PickRobustOTU*, a sequence clustering process will be carried out using Swarm [19] with default configurations in *PickClusterOTU*. Strains with high similarity will be enriched in the same cluster and treated as the same OTU candidate (cOTU). Subsequently, cOTUs will also be picked according to sample ratios and renamed with novel OTU IDs. OTUs would be quantified based on sequence count (*C*) and quantified using the formula ${log}_{2}\left( \frac{C}{N}M+1 \right)$, where *C* represents the sequence count of a specific OTU, *N* represents the total number of sequences, and *M* represents a multiplier coefficient that corrects the minimum $\frac{C}{N}$ into a positive float more than 1. OTU abundance matrix were then normalized by using R package preprocessCore. The final abundance table is a 2-dimensional data matrix with rows corresponding to OTUs and columns corresponding to samples. The workflows of *PickRobustOTU* and *PickClusterOTU* are shown in Additional file 3: Fig. S5. The tool *SampleCorrelation* calculates Pearson’s correlations among samples using the function cor() in R. The tool *SampleClustering* calculates the pairwise distance among all samples using the function dist() and constructs sample clusters using the function hclust() in R. The tool *SamplePCA* implements principle components analysis (PCA) using the function princomp() and visualizes top PCs via the packages scatterplot3d and ggbiplot. The tool *OTUBarplot* explores quasispecies components with bar plots using the R package ggplot2. The tool *OTUNetwork* summarizes normalized weights for each sample and OTU using R scripts and outputs tabular files for Cytoscape-formatted input. The tool *OTUHeatmap* uses the R package gplots to carry out hierarchical clustering analysis. The tool *PhyloTree* provides various substitution models and methods for phylogenetic analysis by interfacing with MEGA CC 6.0 [15] and generates tree files in nwk format. The R package ape is then used for visualizing tree files.

**Useful tools module**

SAMtools [4] is used for file conversion between BAM/SAM files and sorts BAM files by read name or coordinates. Picard tools is used to convert BAM to fastq files. Perl scripts is used in the tool *FastqToFasta* to convert fastq files to fasta files.

**Visualization module**

Circos [20] is used in the tool *Circos* to display quasispecies characteristics in viral genomes. A step-by-step process was developed in the tool *Circos* to guide users to build configuration files for karyotypes and tracks. Integrative Genomics Viewer [21] is used in the tool *IGV* to view the details of genomic data.

**Generation of test data for evaluating QAP**

**Study subjects**

Serum samples from 127 HBV-infected patients were obtained, including 107 antiviral treatment-naïve patients and 20 treatment-experienced patients. Among treatment-naïve patients, 29 immune tolerance (IT) patients, 29 chronic hepatitis B (CHB) patients, 17 liver cirrhosis (LC) patients, 10 acute-on-chronic liver failure (ACLF) and 22 acute hepatitis B (AHB) patients. Among 20 treated patients, there were 10 responders with HBsAg (HBV surface antigen) loss and 10 non-responders with persistent positive HBsAg. All patients were admitted to Shanghai Ruijin Hospital between 2015 to 2017. All patients were diagnosed and treated on the basis of the criteria recommended by the Asian Pacific Association for the Study of Liver (APASL) [22]. Written informed consent according to the Declaration of Helsinki was obtained from each subject. The study protocol was approved by the ethics committee of Ruijin Hospital, Shanghai Jiaotong University, School of Medicine, and all methods were carried out in accordance with the approved guidelines.

**HBV DNA extraction and amplification**

HBV genomic DNA was extracted from 200 μl of serum using a QIAamp UultraSens Virus Kit (Qiagen, Hilden, Germany). For NGS library preparation of HBV DNA, entire HBV genomes were amplified with 10 overlapping fragments by using 10 pairs of primers (Addition file 2: Table S3, and Additional file 3: Fig. S6, primers NP1-NP9). Each HBV fragment was amplified by PCR in 100 µl of buffer containing 30 µl of HBV DNA template, 2 µl of high-fidelity PfuUltra Hotstart DNA polymerase (Agilent Technologies, Santa Clara, CA, USA), corresponding primers and 50 µl of GoTaq Colorless Master Mix (Promega, Madison, WI, USA). PCR parameters were set to 95℃ for 5 minutes, 35 cycles at 95℃ for 15 seconds, 56℃ for 30 seconds, and 72℃ for 30 seconds, with a final extension of 72℃ for 6 minutes. For TGS HBV library preparation, whole HBV genomes were amplified with one pair of primers, designed according to Günther et al. [23] (Additional file2: Table S3, primer TP1). The PCR system was the same with HBV fragment amplification as described previously, but the reaction parameters were different: 95℃ for 15 minutes, 40 cycles at 95℃ for 40 seconds, 56℃ for 30 seconds, and 72℃ for 190 seconds, with a final extension of 72℃ for 7 minutes.

**Clone-based Sanger sequencing of HBV**

Purified whole-genome HBV PCR products were cloned into the pGEM-T (Promega, Madison, WI, USA) vector after the addition of adenylate tails and transformed into *Escherichia coli* strain TOP10 competent cells (Invitrogen) growing on ampicillin plates. An average of 15 positive clones (14-17) per sample were selected and sequenced using ABI 3730 sequencer (Thermo Fisher scientific). After sequencing, raw data were first trimmed based on base quality and assembled using Codon Code Aligner 7.1.1. Multiple alignments were then carried out on all sequences to remove gaps using CLUSTAL X version 2.0 [11]. Recombinant sequences were detected and excluded by RDP3 [24]. Processed sequences were then passed to the tools *ShannonEntropy* and *Diversity* for heterogeneity characterization.

**NGS of HBV**The concentration of PCR products was measured using a Qubit dsDNA HS Assay Kit (Invitrogen). A library of PCR products was established using a Nextera DNA Sample Prep Kit (Illumina, San Diego, CA, USA). Each library was subjected to size selection to remove fragments < 400 bp using AMPure XP beads (Beckman Coulter, Brea, CA, USA) and verified using a Bioanalyzer (Agilent). Before sequencing, each library was quantified by real-time PCR using an NGS Library Quantification Kit (Takara, MountainView, CA, USA). Sequencing of the PCR products was performed using an Illumina Miseq platform, according to the manufacturer’s PE 2 × 300 bp protocol. Image analysis and base calling were performed using Illumina CASAVA software 1.8.2 with default parameters. Raw data were first filtered with the parameters “--readLength” and “--baseQuality 30” and mapped to the reference genome using *MapReadsToRef* with “--program bowtie2”. Sequencing errors were corrected using *ECnQSR* with “--program ShoRAH”. Amplicons were assembled using the tool *AssembleSeq* with the parameters “--ampliconNumbers 10” and “--withIns 1”. Viral haplotype sequences were then passed to ShannonEntropy and Diversity to calculate quasispecies heterogeneities. Quasispecies of all samples were passed to PickRobustOTU for OTU abundance quantification with the parameters “--cutoff 2” and “--ratio 0.05”. Mutations was identified using the tool *MutationCaller* with the parameters “--program gatk,lofreq,varscan”.

**TGS of HBV**

Purified HBV PCR products of each sample were incorporated with unique barcodes, followed by end repair and ligation with universal hairpin adaptors according to the manufacturer’s protocol (Pacific Biosciences, Menlo Park, CA, USA). SMRT sequencing was performed by using P6-C4 chemistry on a PacBio RS II platform (Pacific Biosciences). The raw reads were separated by sample barcodes, and SMRTbell adaptors were then trimmed. Genome filter, assembly and reassembly were performed using tools within SMRT Analysis 2.2.0. Circular consensus sequencer (CCS) reads with a minimum of 6 full passes of the full amplicon (> 20 kb) were selected and generated. CCS reads were passed to the tool *TGSpipeline* and processed with the parameters “--minLen 2500 –maxLen 4000 –errorCorrect Y”. Viral haplotype sequences were then passed to *CutSeqWithIntervals* to extract the sequences of 4 ORFs. Viral heterogeneities were calculated using the tools ShannonEntropy and Diversity.

**HBV simulated data generation**

A simulated data set was generated using Perl scripts with the same proportion of HBV wild-type reference sequences (genotype B, GenBank accession D00329; genotype C, GenBank accession X04615).

**H7N9 cultivation, RNA extraction, amplification and NGS**

The whole procedure was carried out by using published methods [25]. H7N9 influenza viruses were isolated and passaged in Madin-Darby Canine Kidney (MDCK) cells (ATCC Catalogue No. CCL-34) in a biosafety level 3 (BSL-3) laboratory at the Shanghai Public Health Clinical Center. Oseltamivir carboxylate was provided by Hoffmann La-Roche (Switzerland). Two original isolates, i.e., 5190 and 5083, which do not harbour the R292K mutation, were serially passaged in the presence of ascending concentrations of oseltamivir carboxylate (10, 20, 100, 200 and 500 μM) and exogenous neuraminidase (2 mU/ml). The R292K variant was successfully isolated in both strains under these conditions. Viral RNAs were extracted using Trizol LS (Thermo Fisher) and reverse-transcribed using Superscript III (Thermo Fisher) using a cocktail of reverse primers listed in Additional file: Table S7. The full-length HA, NA NP, M and NS segments were amplified by five pairs of specific primers. The PA, PB1 and PB2 segments were each amplified using two pairs of primers using KOD-plus neo (TOYOBO). The amplified products were pooled in equal molar ratios and fragmented using Covaris microTUBE. The sheared DNA was end-repaired and ligated to adaptors using the NEBNext Ultra DNA Library Prep Kit (NEB). The ligated products were subsequently purified and size-selected by AMPure XP beads followed by 6-15 cycles of PCR amplification. The resulting DNA was quantified by Qubit 2.0 and checked by Agilent 2100 using a high-sensitivity DNA chip. Illumina sequencing was performed on an Illumina Hiseq 4000 instrument. Each library generated more than 2G bases.

**H7N9, HCV and HIV NGS data processing**

All raw data was first filtered using *RawDataFiltration* with the parameter “--baseQuality 30” and mapped to the reference genomes (H7N9: CY193251-CY193258; HCV: NC004102; HIV: AF324493 and U39362) using *MapReadsToRef* with the parameter --program bowtie2”. Variations were identified using the tool *MutationCaller* with the parameters “--program gatk,lofreq,varscan”.

**References**

1. S. A: **FastQC: a quality control tool for high throughput sequenc data.** <http://wwwbioinformaticsbabrahamacuk/projects/fastqc/> 2016.

2. Li H, Durbin R: **Fast and accurate long-read alignment with Burrows-Wheeler transform.** *Bioinformatics* 2010, **26:**589-595.

3. Langmead B, Salzberg SL: **Fast gapped-read alignment with Bowtie 2.** *Nat Methods* 2012, **9:**357-359.

4. Li H, Handsaker B, Wysoker A, Fennell T, Ruan J, Homer N, Marth G, Abecasis G, Durbin R: **The Sequence Alignment/Map format and SAMtools.** *Bioinformatics* 2009, **25:**2078-2079.

5. Baaijens JA, Aabidine AZE, Rivals E, Schonhuth A: **De novo assembly of viral quasispecies using overlap graphs.** *Genome Res* 2017, **27:**835-848.

6. Zagordi O, Bhattacharya A, Eriksson N, Beerenwinkel N: **ShoRAH: estimating the genetic diversity of a mixed sample from next-generation sequencing data.** *BMC Bioinformatics* 2011, **12:**119.

7. Prabhakaran S, Rey M, Zagordi O, Beerenwinkel N, Roth V: **HIV Haplotype Inference Using a Propagating Dirichlet Process Mixture Model.** *IEEE/ACM Trans Comput Biol Bioinform* 2014, **11:**182-191.

8. Jayasundara D, Saeed I, Maheswararajah S, Chang BC, Tang SL, Halgamuge SK: **ViQuaS: an improved reconstruction pipeline for viral quasispecies spectra generated by next-generation sequencing.** *Bioinformatics* 2015, **31:**886-896.

9. Prosperi MC, Salemi M: **QuRe: software for viral quasispecies reconstruction from next-generation sequencing data.** *Bioinformatics* 2012, **28:**132-133.

10. Camacho C, Coulouris G, Avagyan V, Ma N, Papadopoulos J, Bealer K, Madden TL: **BLAST+: architecture and applications.** *BMC Bioinformatics* 2009, **10:**421.

11. Larkin MA, Blackshields G, Brown NP, Chenna R, McGettigan PA, McWilliam H, Valentin F, Wallace IM, Wilm A, Lopez R, et al: **Clustal W and Clustal X version 2.0.** *Bioinformatics* 2007, **23:**2947-2948.

12. Edgar RC: **MUSCLE: multiple sequence alignment with high accuracy and high throughput.** *Nucleic Acids Res* 2004, **32:**1792-1797.

13. Sievers F, Higgins DG: **Clustal Omega, accurate alignment of very large numbers of sequences.** *Methods Mol Biol* 2014, **1079:**105-116.

14. Crooks GE, Hon G, Chandonia JM, Brenner SE: **WebLogo: a sequence logo generator.** *Genome Res* 2004, **14:**1188-1190.

15. Kumar S, Stecher G, Tamura K: **MEGA7: Molecular Evolutionary Genetics Analysis Version 7.0 for Bigger Datasets.** *Mol Biol Evol* 2016, **33:**1870-1874.

16. McKenna A, Hanna M, Banks E, Sivachenko A, Cibulskis K, Kernytsky A, Garimella K, Altshuler D, Gabriel S, Daly M, DePristo MA: **The Genome Analysis Toolkit: a MapReduce framework for analyzing next-generation DNA sequencing data.** *Genome Res* 2010, **20:**1297-1303.

17. Koboldt DC, Zhang Q, Larson DE, Shen D, McLellan MD, Lin L, Miller CA, Mardis ER, Ding L, Wilson RK: **VarScan 2: somatic mutation and copy number alteration discovery in cancer by exome sequencing.** *Genome Res* 2012, **22:**568-576.

18. Wilm A, Aw PP, Bertrand D, Yeo GH, Ong SH, Wong CH, Khor CC, Petric R, Hibberd ML, Nagarajan N: **LoFreq: a sequence-quality aware, ultra-sensitive variant caller for uncovering cell-population heterogeneity from high-throughput sequencing datasets.** *Nucleic Acids Res* 2012, **40:**11189-11201.

19. Mahe F, Rognes T, Quince C, de Vargas C, Dunthorn M: **Swarm v2: highly-scalable and high-resolution amplicon clustering.** *PeerJ* 2015, **3:**e1420.

20. Krzywinski M, Schein J, Birol I, Connors J, Gascoyne R, Horsman D, Jones SJ, Marra MA: **Circos: an information aesthetic for comparative genomics.** *Genome Res* 2009, **19:**1639-1645.

21. Robinson JT, Thorvaldsdottir H, Winckler W, Guttman M, Lander ES, Getz G, Mesirov JP: **Integrative genomics viewer.** *Nat Biotechnol* 2011, **29:**24-26.

22. Sarin SK, Kumar M, Lau GK, Abbas Z, Chan HL, Chen CJ, Chen DS, Chen HL, Chen PJ, Chien RN, et al: **Asian-Pacific clinical practice guidelines on the management of hepatitis B: a 2015 update.** *Hepatol Int* 2016, **10:**1-98.

23. Gunther S, Li BC, Miska S, Kruger DH, Meisel H, Will H: **A novel method for efficient amplification of whole hepatitis B virus genomes permits rapid functional analysis and reveals deletion mutants in immunosuppressed patients.** *J Virol* 1995, **69:**5437-5444.

24. Martin DP, Lemey P, Lott M, Moulton V, Posada D, Lefeuvre P: **RDP3: a flexible and fast computer program for analyzing recombination.** *Bioinformatics* 2010, **26:**2462-2463.

25. Zhang X, Song Z, He J, Yen HL, Li J, Zhu Z, Tian D, Wang W, Xu L, Guan W, et al: **Drug susceptibility profile and pathogenicity of H7N9 influenza virus (Anhui1 lineage) with R292K substitution.** *Emerg Microbes Infect* 2014, **3:**e78.
