# Supplementary material for: An integrated software for virus community sequencing data analysis
Source: BMC Genomics. 2020 May 15;21:363. doi: 10.1186/s12864-020-6744-4 (PMC7227348; doi:10.1186/s12864-020-6744-4)
Supplement: Supplementary file 3 — Additional file 3 Figure. S1. Screenshot of the QAP main program in command line. Figure S2. Schematic overview of the tool TGSpipeline. Figure S3. Schematic overview of the tool FixCircRef. Figure S4. Example results generated by QAP. Figure S5. Schematic overview of OTU picking. Figure S6. Example results generated by Circos and IGV tools showing the amplicons of HBV whole-genome sequencing. Figure S7. Bland-Altman analysis of heterogeneity of 4 ORFs in TGS data compared with CBS data. [file 12864_2020_6744_MOESM3_ESM.docx]

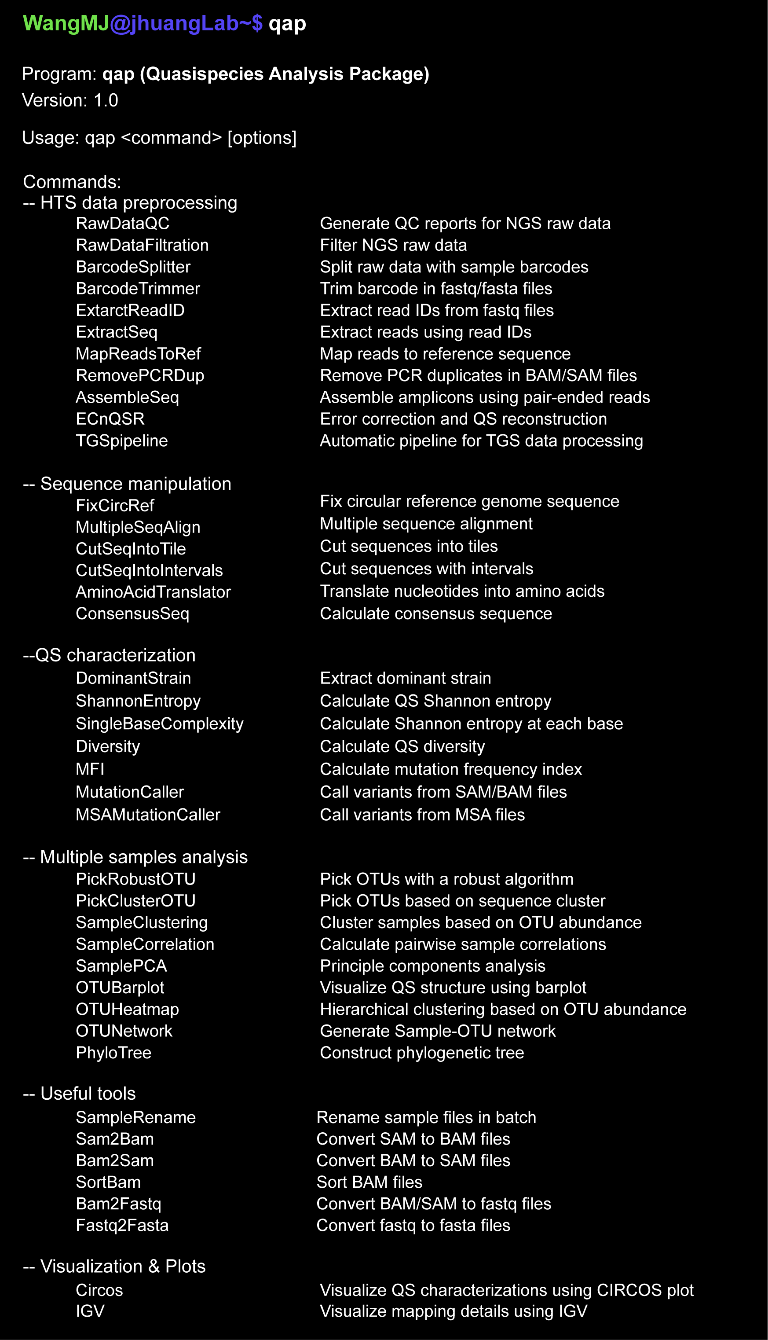


**Fig. S1 Screenshot of the QAP main program in command line.** Run command “qap” in the shell, and the program will output welcome information.


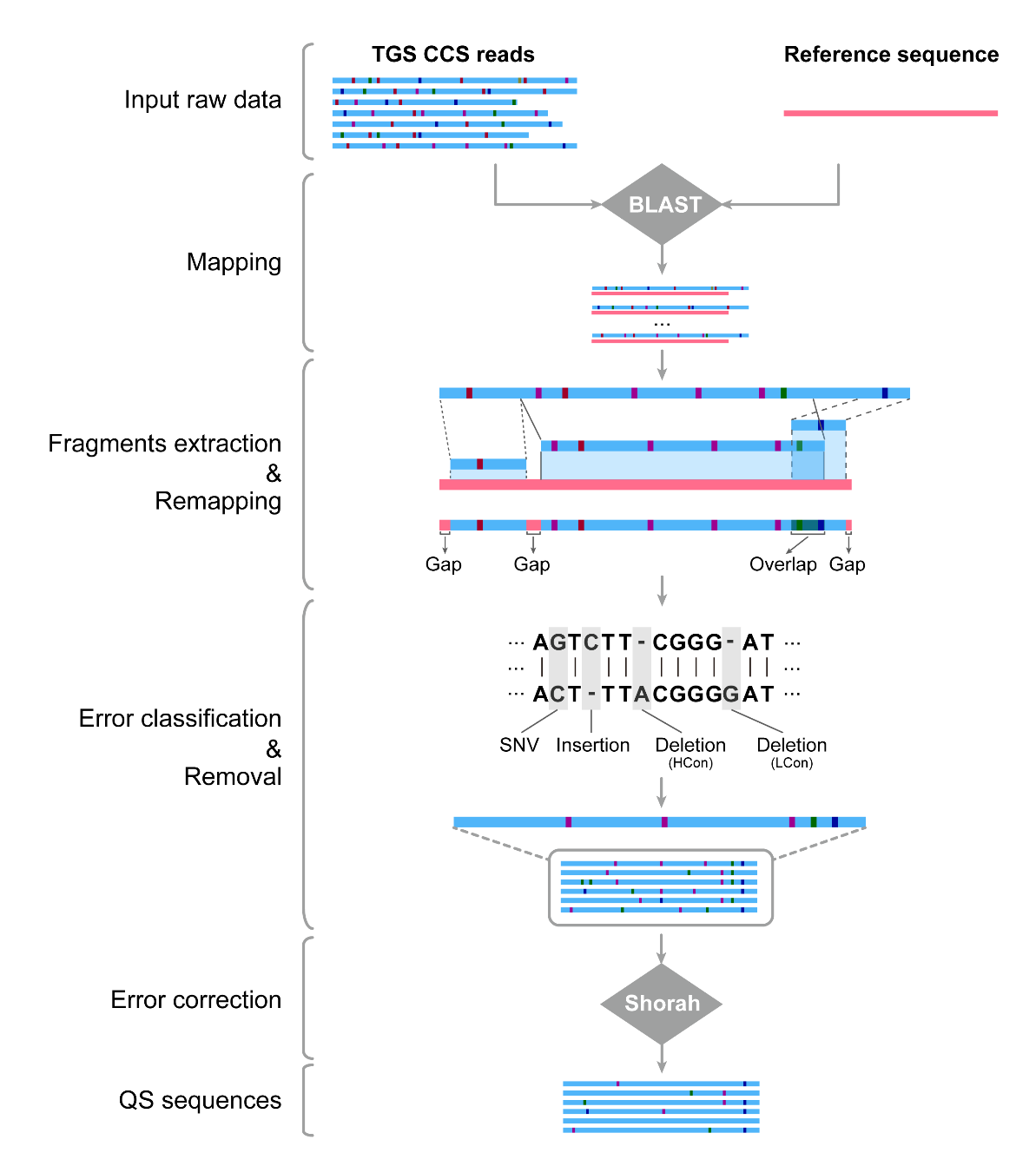


**Fig. S2 Schematic overview of the tool TGSpipeline.** The data processing workflow applied in the TGSpipeline could be separated into 6 steps (marked on the left). Reads are coloured in blue, and the reference sequence is coloured in red. Scattered dots with different colours represent sequencing errors and variations. The rhombi represent the programs used. Arrows indicate the flow between input, processes and output.


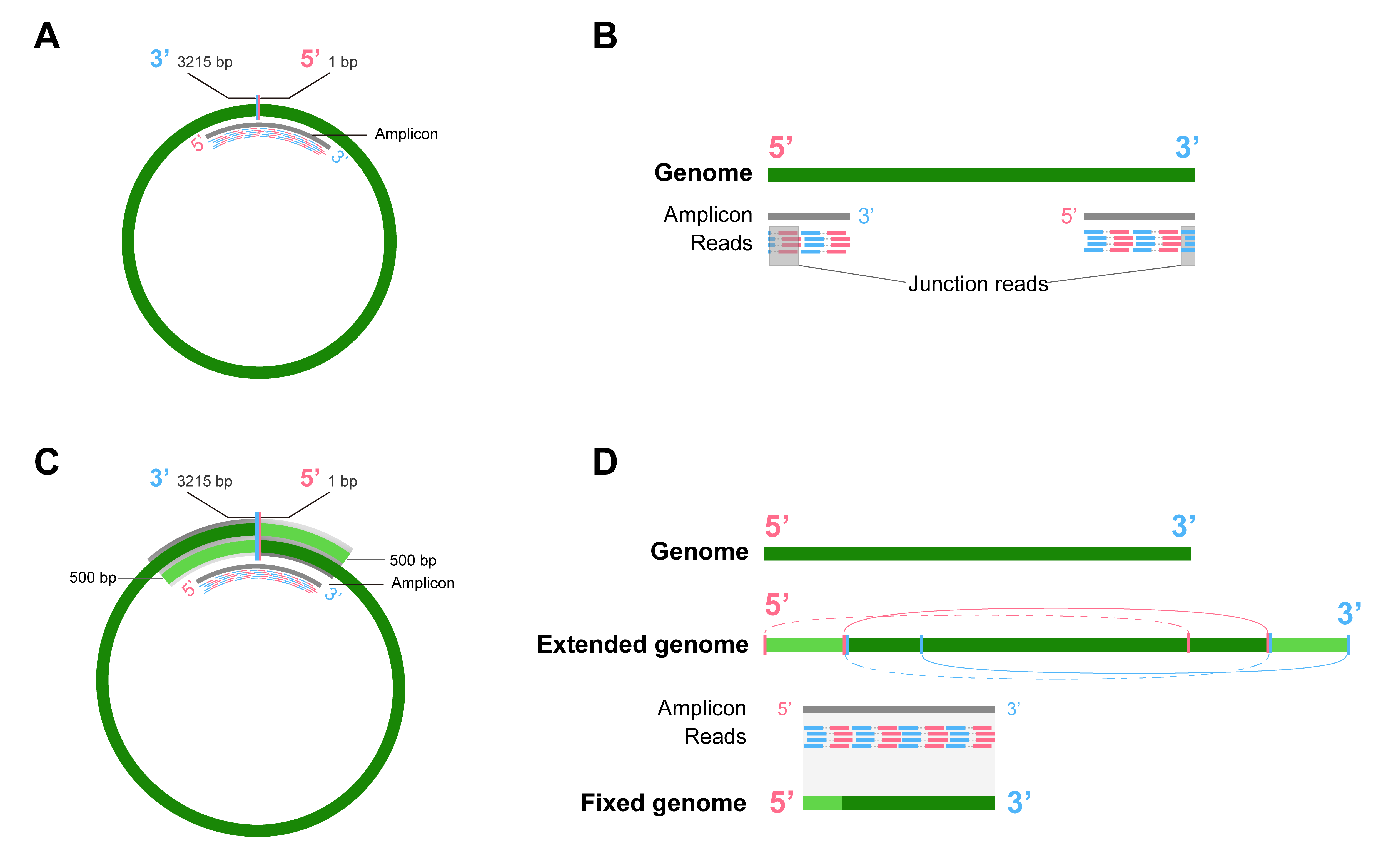


**Fig. S3 Schematic overview of the tool FixCircRef.** Green thick lines (both circle and straight) represent the viral genome with 5’ ends highlighted in red and 3’ ends highlighted in blue. Grey medium-thick lines represent sequencing amplicons. Short lines coloured in red and blue represent paired reads. (A) A circular viral genome with an amplicon across both ends. (B) A straightened circular reference genome with a truncated amplicon and junction reads. (C) A circular viral genome with 500-bp extensions on both ends. (D) A straightened extended circular reference genome with genomes and reads mapped to extended regions. The red dotted thin line and red solid thin line in the extended genome mark the 5’ and 3’ ends of the extended region and its original region (same with blue thin lines below the extended genome).


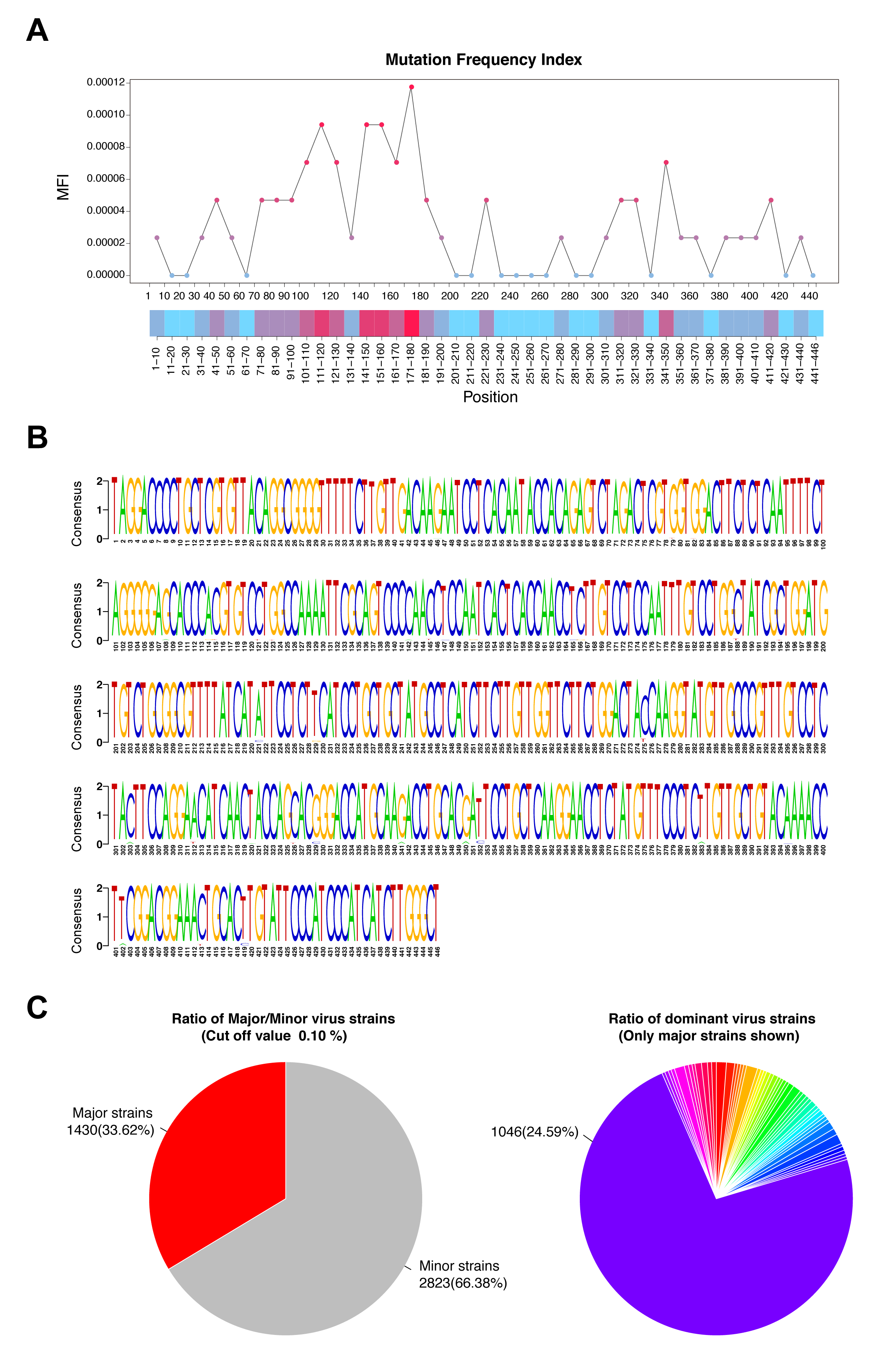


**Fig. S4 Example results generated by QAP.** (A) Plots generated by the tool DominantStrain. The ratios of major strains and minor strains are shown by the pie chart in the left panel, and the ratio of the dominant strain within major strains is shown by the pie chart in the right panel. Raw counts and proportion values are labelled in the plots. (B) A Weblogo plot generated by the tool ConsensusSeq. Bases are represented using different colours, and the letter heights correspond to the relative ratios of each base. **(C)** Plots generated by the tool MFI. MFI values of continuous fragments are represented using both the dotted line in the upper panel and the heat map in the lower panel. Dot and heat map colours correspond to MFI values; red colour means high, and blue means low. Position intervals are also labelled in the plots.


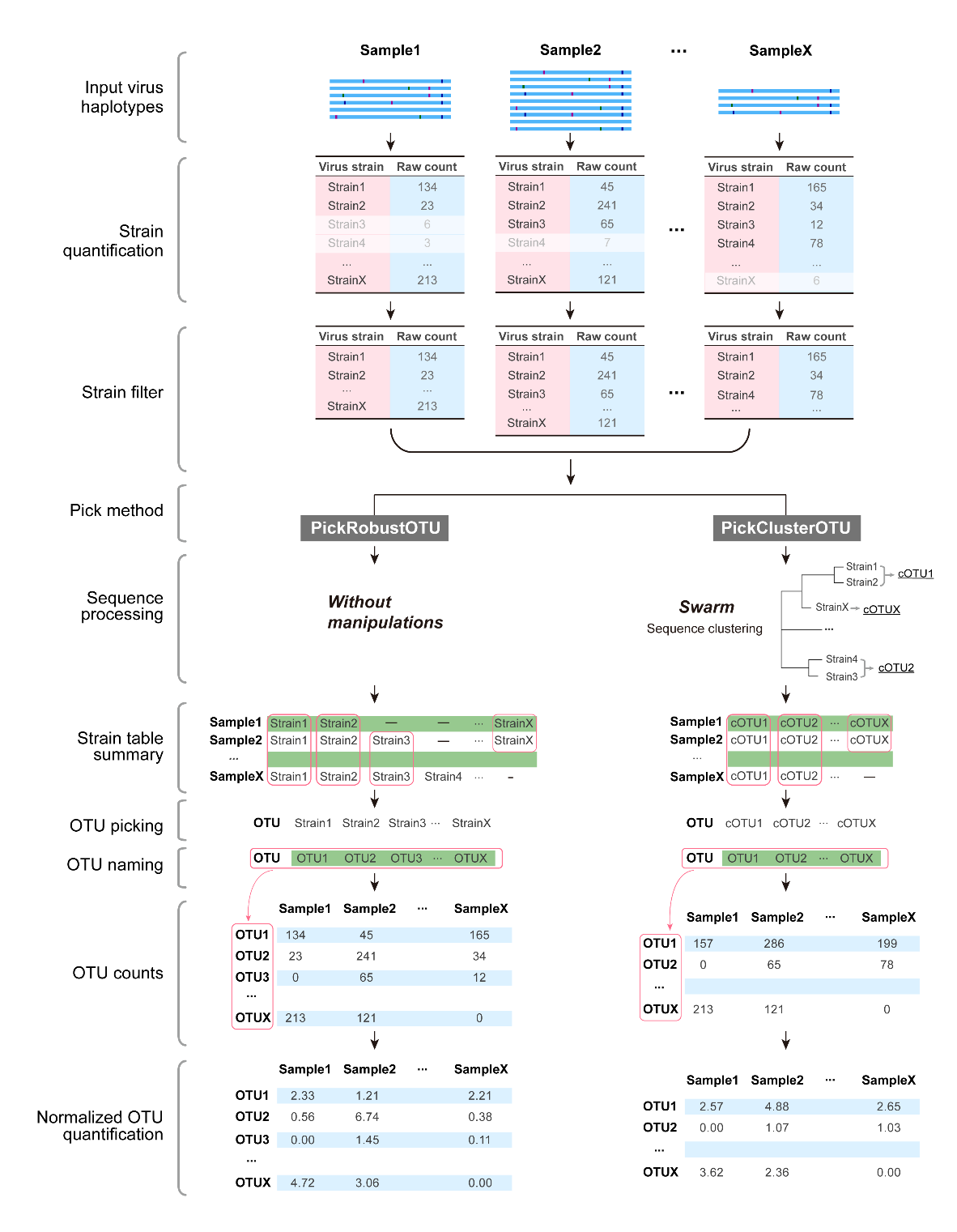


**Fig. S5 Schematic overview of OTU picking.** The data processing workflow applied in OTU picking can be separated into 10 steps (marked on the left). Blue lines represent sequencing reads, and scattered dots with different colours represent variations. Arrows indicate the flow between input, processed and output. The programs used are highlighted in bold italic font. Strains with low counts in strain quantification step are marked with transparent background. Strains and OTUs are highlighted with red rectangles.


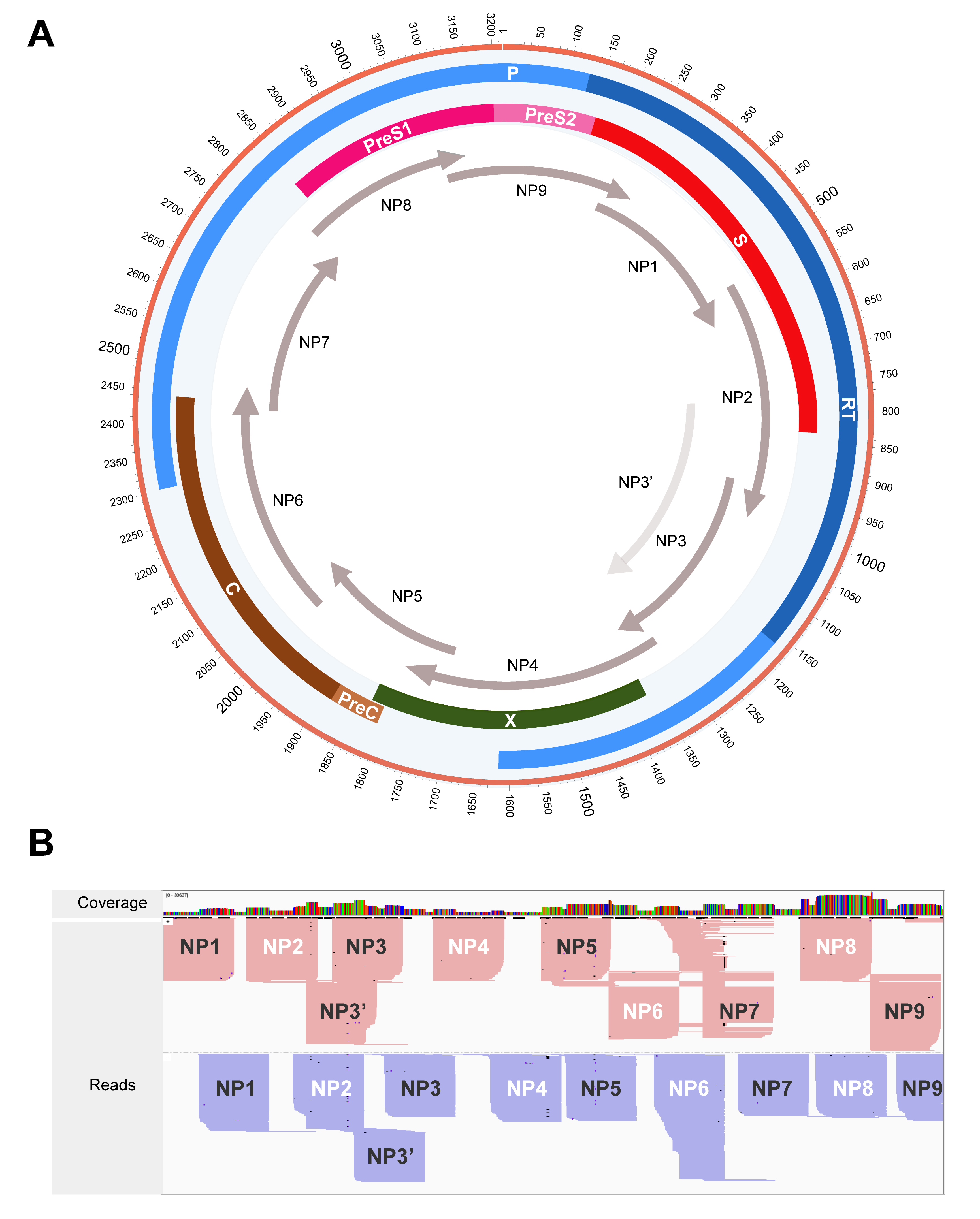


**Fig. S6 Example results generated by Circos and IGV tools showing the amplicons of HBV whole-genome sequencing.** (A) Plot generated by the tool Circos showing the locations of 10 amplicons in NGS of HBV quasispecies (modified from Circos output). The outer track shows the positions of the HBV genome, and the inner arcs with different colours correspond to the main functional regions in the HBV genome. Amplicons are represented by arrows with their names labelled. (B) Plot generated by the tool IGV showing the mapping details of sample RJ001 for demonstration (modified from IGV output). The upper panel shows the sequencing coverage and depth. The lower panel shows a condensed overview of all mapped reads with paired reads highlighted with different colours (blue and red). The names of all amplicons are also marked in the plot.


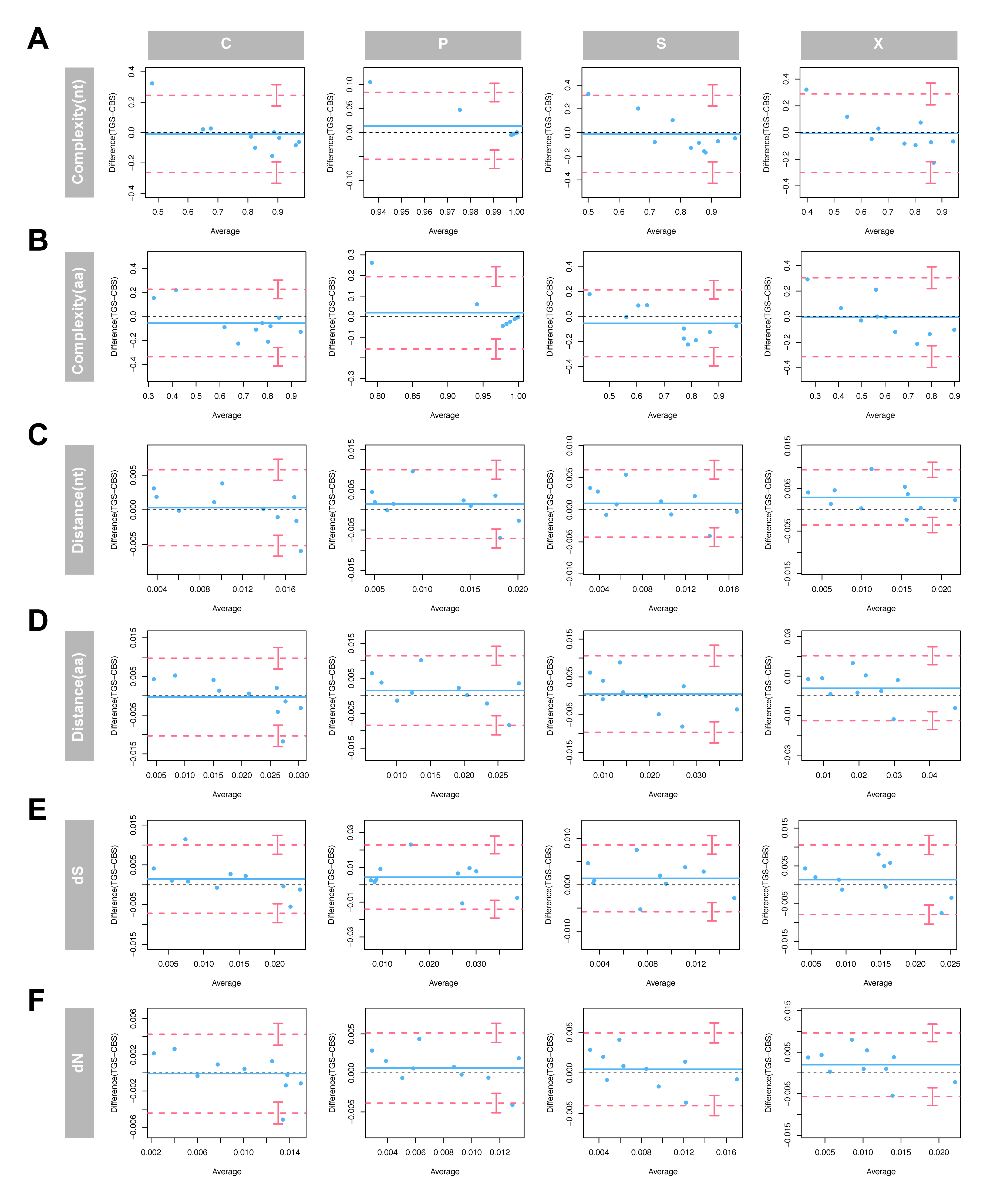


**Fig. S7 Bland-Altman analysis of heterogeneity of 4 ORFs in TGS data compared with CBS data.** (A) Complexity at the nucleotide level for the C, P, S, and X genes, respectively. (From right panel to left panel, the same below) (B) Complexity at the amino acid level. (C) Mean genetic distance at the nucleotide level. (D) Mean genetic distance at the amino acid level. (E) dS. (F) dN. The blue horizontal lines were drawn at the mean difference; the red dotted horizontal lines were drawn at the upper and lower limits of agreement, which were defined as the mean difference ± 1.96 × SD (standard deviation) of the difference. The short vertical lines indicate the 95% consistency limit of the upper and lower bounds of agreement. Values within the limits are shown as blue dots.
